# Clinical Outcomes of Descemet’s Membrane Endothelial Keratoplasty without Routine Prophylactic Peripheral Iridotomy

**DOI:** 10.3390/vision7020041

**Published:** 2023-05-19

**Authors:** Ritika Mukhija, Gabriella Quiney, Mayank A. Nanavaty

**Affiliations:** 1 Sussex Eye Hospital, University Hospitals Sussex NHS Foundation Trust, Eastern Road, Brighton BN11 2DH, UK; 2 Brighton & Sussex Medical School, University of Sussex, Falmer, Brighton BN1 9RH, UK

**Keywords:** Descemet’s membrane endothelial keratoplasty, DMEK, Fuchs, peripheral iridotomy, pupillary block

## Abstract

**Objectives:** To analyze the outcomes and complications of Descemet‘s membrane endothelial keratoplasty (DMEK) performed without prophylactic peripheral iridotomy (PI). **Methods**: Design: Retrospective study. Setting: Institutional, tertiary care eye hospital. Study Population: All patients who underwent DMEK or DMEK combined with phacoemulsification (DMEK triple) for Fuchs endothelial dystrophy, using a standardized protocol between August 2016 and July 2021, were included. Previous glaucoma surgery, laser PI, aphakia, or complicated pseudophakia were excluded. Main outcome measures: Primary outcomes: Incidence of pupillary block (PB). Secondary outcomes: Graft detachment (GD), rebubbling rates, uncorrected (UCDVA) and best corrected logMAR distance visual acuity (BCDVA), and endothelial cell loss (ECL) at six months. Data were analyzed using the chi-square test and stepwise backward regression analysis. **Results**: 104 eyes of 72 patients were included. Four eyes (3.8%) developed PB; in two of these cases, standard protocol was not followed. Overall minor GD occurred in 43.2% (n = 45); significant GD was present only in 7 eyes (6.6%). Overall slit lamp rebubbling rate was 30% (n = 35), though only four patients were rebubbled in theatre (3.8%). PB, GD, and rebubbling rates did not vary with the surgeon, surgery, or tamponade (air or SF6 gas). UCDVA, BCDVA, and ECL at 6 months were 0.29 ± 0.31, 0.20 ± 0.28, and 40.46 ± 20.36%, respectively. **Conclusions**: Compared to previously reported outcomes of DMEK with PI, our results of PI less DMEK using a standardized protocol have a similar incidence of pupillary block, graft detachment, and rebubbling, with comparable visual acuity and endothelial cell loss.

## 1. Introduction

The advent of Descemet’s membrane endothelial keratoplasty (DMEK) has revolutionized the standard of care for corneal endothelial disease and it is now well established that DMEK provides faster and visual rehabilitation than other endothelial keratoplasty procedures such as Descemet’s stripping automated endothelial keratoplasty (DSAEK), Descemet’s stripping endothelial keratoplasty (DSEK), and penetrating keratoplasty (PK) [1,2,3]. However, graft detachment remains one of the most frequent complications and challenges with DMEK. To avoid this, the anterior chamber is filled with either air or gas at the end of surgery to provide tamponade [4,5]. While this is crucial for graft attachment, it can result in pupillary block ocular hypertension—one of the most feared complications in the early postoperative period after DMEK. To prevent this, surgeons often perform prophylactic peripheral iridotomy either before, using the Nd-YAG laser, or during the procedure, using surgical iridectomy; however, despite that, air or gas often needs to be released [6,7,8].

We have been routinely performing DMEK without preoperative PI or intraoperative surgical iridectomy using a standardized protocol for many years. This retrospective study aims to analyze whether avoiding laser iridotomy or surgical iridectomy affects the rates of pupillary block, graft detachment, graft failure, and endothelial cell loss compared to those documented in the literature.

## 2. Materials and Methods

This study is designed as a single-center, retrospective, interventional, consecutive case series. It was approved by the Clinical Audit Committee at Sussex Eye Hospital, University Hospitals Sussex NHS Foundation Trust (registration number 4735) and was performed as per the tenets of the Declaration of Helsinki. Informed consent to collect data for audit purposes was obtained from the patients before surgery as part of routine clinical practice.

A list of all transplants performed under a single surgeon-led team was retrieved from the corneal transplant coordinator at the hospital. All eyes undergoing DMEK or DMEK in combination with phacoemulsification surgery (DMEK triple) for Fuchs endothelial dystrophy only, either performed or supervised by a single surgeon (MAN) using standardized surgical technique and postoperative care between August 2016 and July 2021, were included. Eyes with a history of previous glaucoma surgery which required a surgical iridectomy or previous Nd-YAG laser iridotomy and aphakic eyes or eyes where the intraocular lens was not in the capsular bag were excluded. For this study, surgical iridectomy and laser iridotomy will be referred to as PI.

The primary outcome measure was the incidence of pupillary block (PB). Both primary pupillary block (Type I: bubble pushing the pupil against the lens restricting any aqueous movement through the pupil) and secondary angle closure (Type II: bubble misdirection resulting in mechanical angle closure) were accounted for as a pupillary block for analysis. Secondary outcome measures were graft detachment (GD), rebubbling rates, uncorrected logMAR distance visual acuity (UCDVA), best corrected logMAR distance visual acuity (BCDVA), endothelial cell loss (ECL), and graft survival at six months. Graft detachment was assessed clinically and on an anterior segment OCT. It was recorded as either mild (involving less than one-third of the peripheral graft area) or significant (involving more than one-third of the graft area or any detachment involving the central visual axis) [4,7].

### 2.1. Surgical Technique

All grafts were prepared by the surgeon in the theatre before the surgery on the same day using a manual dissection technique with modifications to the originally described method by Melles [9]. Following trephination and creation of a triangular mark to identify the correct orientation [10], they were transferred into a single-use cartridge connected to tubing and syringe (Geuder injector system; German Geuder AG, Heidelberg, Germany) just before transplantation. After implantation and unfolding of the donor tissue, the graft was attached to the recipient’s stroma with a complete anterior chamber filling of either air or 50% sulphur hexafluoride (SF6). The main incision and two paracenteses were sutured using 10- monofilament nylon. Another 5 o’clock paracentesis was made as a vent incision for aqueous and air/gas release on a slit lamp, as previously described by the author [11].

### 2.2. Standardized Postoperative Follow-Up

All patients were reviewed on the ward one to two hours after surgery on postoperative day 0 (POD-0) and a small amount of air or gas was released using the 5 o’clock paracentesis [11]. This was performed to ensure that the anterior chamber depth returned to normal on slit lamp assessment with a tamponade bubble in front of the pupil and approximately 80% of the anterior chamber volume. Subsequently, some aqueous was released using the same incision on postoperative day 1 (POD-1) to ensure that anterior chamber volume is reset to standard with the tamponade bubble occupying approximately 70% of the anterior chamber. This is specifically important with SF6 gas, as we expect gas bubble expansion with the deepening of the AC in the initial post-operative period; whereas with air, the idea is to make space in the AC to account for continually producing aqueous whilst the air bubble shrinks in a few days. Both these procedures were performed by the surgeon at the slit-lamp under topical anaesthesia and standard aseptic precautions (G. Proxymetacaine 0.5% minims and G. Povidone-iodine minims, Bausch & Lomb, Laval, QC, Canada) [11]. We do not routinely measure intraocular pressure at the end of procedure or during tamponade release because IOP measurement can be unreliable due to variable pachymetry; instead, we rely on visible resolution of epithelial oedema along with reduction in anterior chamber depth to assess IOP.

The patients were then reviewed after one week. They were advised to lie supine, when possible, for the first three days; however, strict positioning was not advocated. Postoperative medications included topical antibiotic steroid combination (G. Tobradex, Novartis Pharmaceuticals, Basel, Switzerland) four times a day for four weeks and topical cycloplegia (G. Cyclopentolate 1% minims, Bausch & Lomb) three times a day and IOP lowering (oral acetazolamide 250 mg sustained release twice a day) for the first week only. Sutures were removed in the first four weeks, and patients were switched to a plain topical steroid (G. Lotemax, Bausch & Lomb, four times a day for three months, twice a day for three months, and one a day there on until 2 years).

### 2.3. Data Analysis

Eligibility was determined by a single ophthalmologist (RM) after assessing each patient’s notes and the local electronic patient record (MediSoft Limited, Leeds, UK). Data were recorded on a spreadsheet (Excel, Microsoft^®^ Inc, Redmond, WA, USA). Data were analyzed using Statplus mac software (Version 7.7.11, AnalystSoft Inc., Alexandria, VA, USA) and presented as mean ± standard deviation. After testing the normality of the data, *t*-test was used to compare UCDVA, BCDVA, and ECL between grades of the surgeon, surgery (DMEK triple or DMEK), tamponade (air or SF6), graft detachment, and rebubbling in groups with or without PB between 1 and 6 months. A chi-square test was used to assess the effect of the surgeon grade, surgery, tamponade, graft detachment, and rebubbling. Stepwise backward logistic regression analysis was performed to look at the factors responsible for PB. A *p* value < 0.05 was considered significant.

## 3. Results

A total of 113 DMEK surgeries were performed under a single corneal surgeon-led team for Fuchs endothelial dystrophy from August 2016 to June 2021. Of these, nine eyes were excluded (previous PI or glaucoma surgery, complicated pseudophakia with PBK with unknown previous surgical history elsewhere), and 104 of 72 patients (M:F = 34:38) were included for analysis. The average age in this study was 71.13 ± 10.41years (range: 44 to 88 years). Baseline parameters are summarized in Table 1.

### 3.1. Primary Outcome: Pupillary Block

Four patients (3.8%) developed pupillary block (PB); one was a type I, and the other three were a type II pupillary block (Table 2). Interestingly, in two of these cases of PB, the standard protocol was not followed; in the other two it is possible that there was inadequate release of gas and aqueous on day 1. Overall, eight patients (air: n = 7; gas: n = 1) did not have tamponade release on the day of surgery; of these, four patients with air did not require any release as documented in notes (bubble meniscus was already around 80% when reviewed after surgery), three patients had missing documentation on notes (air: n = 2; gas: n = 1) but had no events in post-operative period, and one developed type 1 PB. At day 1, 16 patients (air: n = 15; gas: n = 1) did not have any aqueous release; of these, 1 patient with gas developed type 2 PB later, 12 patients with air did not require any release, and 3 patients did not have any documentation in notes.

There was no difference in the incidence of the PB when compared between two different grades of the surgeon (*p* = 0.44), type of surgery (*p* = 0.14), or tamponade (*p* = 0.80). A stepwise backward regression analysis showed that PB was negatively related to slit-lamp release on POD-1 (i.e., no release performed on slit-lamp at POD-1) and positively related to BCDVA at 1 month (i.e., higher logMAR BCDVA value/poorer BCDVA at 1 month) but not BCDVA at 6 months, which was comparable.

### 3.2. Secondary Outcomes

(a) Graft detachment and rebubbling:

A total of 45 eyes (43.2%) had graft detachment; of these, 38 had mild detachment (36.5%) and 7 were significant (6.7%).

(1) Mild detachments (36.5%): Of the 38 eyes with mild detachment (less than one third), about 10 had inferior detachment, which settled conservatively. A top-up injection of tamponade (air) was performed on a slit-lamp for the remaining 28.

(2) Significant detachment (6.7%): Four of the seven significant detachments were bubbled in theatre; the other three were shallow central detachments that settled with air injection on the slit-lamp. Of the four eyes rebubbled in theatre, two had mild folding of the graft edge inferiorly, one had partial dislocation and rolling of graft, and one had small detachment involving visual axis but was unable to cooperate on the slit-lamp.

Graft detachment rates were statistically similar between type of surgery (*p* = 0.41) and surgeon grades (*p* = 0.06; overall GD: n = 32 out of 64 for consultant and 12 out of 40 for fellow). Further, there was no significant difference in GD rates with type of tamponade (*p* = 0.33); however, nearly half the eyes with air as a tamponade had mild graft detachment (13 out of 27) as compared to one-third with gas (25 out of 77). Similarly, there was no difference in slit lamp or theatre rebubbling rates when compared between two different grades of the surgeon (*p* = 0.09 and 0.57), type of surgery (*p* = 0.10 and 0.96), or tamponade (*p* = 0.38 and 0.96), respectively. Twenty-one and three eyes out of a total sixty-four that were operated on by a consultant required slit lamp and theatre rebubbling, whereas seven and one eye out of forty eyes operated by a fellow required the same. More complex cases were performed by consultants and routine cases by fellow.

(b) Visual acuity:

At 1 and 6 months, UCDVA were 0.61 ± 0.56 logMAR (range: 3 to 0 logMAR) and 0.29 ± 0.31 logMAR (range: 2 to −0.12 logMAR) and BCDVA were 0.43 ± 0.61 logMAR (range: 3 to −0.12 logMAR) and 0.20 ± 0.28 (range: 2 to −0.12 logMAR), respectively. There was no significant difference in UCDVA and BCDVA with grade of surgeon at 1 (*p* = 0.32 and 0.21) and 6 months (*p* = 0.38 and 0.48), respectively. Likewise, there was no significant difference in UCDVA and BCDVA between DMEK Vs DMEK triple at one (*p* = 0.26 and 0.13) month, but both visual acuities were significantly better in the DMEK triple group at six months (UCDVA: 0.24 ± 0.23; BCDVA: 0.15 ± 0.16) as compared to DMEK group (UCDVA: 0.29 ± 0.33; BCDVA: 0.33 ± 0.45) (*p <* 0.01 and *p* < 0.01, respectively).

There was no difference in UCDVA and BCDVA with the type of tamponade at one (*p* = 0.28 and *p* = 0.29) and six months (*p* = 0.10 and *p* = 0. 4). As can be expected, both UCDVA and BCDVA at one month were significantly better in eyes with no GD compared to eyes with mild or significant GD (*p* < 0.01 and *p* < 0.01); this difference was seen at six months as well (*p* = 0.04 and *p* = 0.02). Further, eyes requiring rebubbling had significantly worse UCDVA and BCDVA at one month compared to eyes that did not require rebubbling (*p* = 0.00 and *p* < 0.01); again, this difference was noted at six months (*p* = 0.04 and *p* < 0.01). For eyes with or without PB, there was no difference in UCDVA and BCDVA at one month (*p* = 0.35 and *p* = 0.29) and six months (*p* = 0.26 and *p* = 0.49).

(c) ECL and graft survival:

Average ECL at six months was 40.46 ± 20.36% (1.87 to 79.1%). This did not differ between type of surgery (*p* = 0.14) or whether GD was present (*p* = 0.28), type of tamponade, and rebubbling performed (*p* = 0.26), and was just short of reaching statistical significance with higher ECL when a fellow performed surgery as compared to the consultant (*p* = 0. 5). There was insufficient data to assess significance compared with the type of tamponade and incidence of PB. ECL at six months was present for only one case with PB, and it measured 1449 cells/mm^2^ with an ECL of 44%.

(d) Complications:

Two eyes required more than one rebubbling (twice and thrice, respectively) and were labeled as primary graft failure. They underwent secondary DSAEK subsequently; one of the cases had type II PB. There were no cases of endothelial failure within six months of follow up. Three eyes developed cystoid macular oedema. One patient was a steroid responder and required anti-glaucoma medications to control intraocular pressure. One patient required an intra-ocular (IOL) exchange four months after DMEK because of opacification of hydrophilic acrylic IOL with SF6 gas. One patient developed endophthalmitis one week after suture removal and five weeks after DMEK necessitating vitrectomy and had a final BCDVA of logMAR 0.3 (or 6/12) at six months.

## 4. Discussion

Our study of 104 eyes reports real-world outcomes of DMEK performed without routine prophylactic PI. Many surgeons continue to perform either preoperative or intraoperative PI to prevent pupillary block following tamponade in DMEK [7,8,12,13,14,15,16]. However, despite this, PB may occur and a further release of tamponade is often required [8]. Unfortunately, many studies documenting outcomes of DMEK do not routinely mention the incidence of PB. Nonetheless, our rate of 3.8% is well within the reported rates in the literature, varying from 0 to 15% [8]. (Table 3) Performing a PI, albeit a simple procedure, is not without risks. It can result in glare, photophobia, monocular diplopia, and even damage lens zonules. Further, preoperative PI may be difficult owing to poor visibility and implies an additional visit to the hospital. Intraoperative PI may result in bleeding or fibrin formation, impairing graft adherence and success [17].

Livny et al. reported outcomes of DMEK without routine PI in a retrospective case series of 31 eyes. They followed a similar approach, filling the anterior chamber with 20% SF6 and releasing the same to two-thirds of the volume approximately 90 min after the procedure. They reported no case of pupillary block in their study [24]. The reported graft detachment and rebubbling rates were 32% and 16%, respectively [19]. Röck et al. studied the incidence and risk factors of PB caused by an air bubble in the early postoperative period after DMEK. In their retrospective study of 306 eyes, 30 eyes (9.8%), all with iridectomy at the 12 o’clock position showed a postoperative IOP elevation within the first postoperative day, while there was no PB in eyes with inferior PI. For the former, 25 eyes (8.2%) had PB from air anterior to iris (type I) and 5 eyes (1.6%) had angle closure from air migration posterior to the iris (type II) [19]. To prevent a tamponade-related pupillary block, PI should ideally be performed inferiorly. Considering superior PI as ineffective, this rate of 9.8% PB is higher than 3.8% in our study. Moreover, in our study, two out of four cases of PB happened in eyes where our standard protocol was not followed. It is, however, important to note that the definition of PB can vary amongst studies and between phakic and pseudophakic eyes after DMEK. In our study, there were no cases of phakic DMEK, as all phakic eyes had DMEK in combination with phacoemulsification.

Our graft detachment rates, both overall (43%) and clinically significant GD rate (<7%), are well within the range documented between 4 and 56% in the literature. Fortunately, not all significant detachments had to be rebubbled in the theatre, and only four eyes needed another visit, with a rebubbling rate of less than 4% (n = 4; 3.8%) in our study. Our overall rebubbling rates, including top-up injection of tamponade on slit lamp, was 30%, which is also within the reported rates of rebubbling in the literature varying between 0 and 76%, and closer to the mean rate of 28.8% [25]. As there are no standard definitions, graft detachment and rebubbling rates vary between studies. We also noted that our threshold for slit-lamp air injection for mild detachments was much lower in the earlier cohort of eyes. With more experience and evidence that most small or inferior detachments settle conservatively, we now observe these than reinjecting air on slit-lamp [26]. Further analysis of our recent 40 cases performed in the last three years (August 2018 to July 2021) revealed that 11 eyes had mild detachment, and 5 received an anterior chamber air injection on a slit-lamp. This contrasted with 64 older cases (August 2016 to July 2018), where 27 eyes had mild detachment, and 21 received anterior chamber air injection on the slit-lamp.

We did not find a significant difference in GD and rebubbling rates based on the type of tamponade; however, nearly half of the eyes with air had visually insignificant GD (13 of 27 eyes) as compared to less than one-third (25 of 77 eyes) when SF6 gas was used as a tamponade. Similar trends were seen for rebubbling, as reported previously in the literature [22]. In a fellow eye comparison study, authors noted that 13 of 68 eyes (19.1%) with an air tamponade needed rebubbling as compared to 4 of 68 eyes (5.9%) with an SF6 gas tamponade (*p* = 0.04) [22]. In a metanalysis comparing 100% air with 20% SF6 in DMEK, authors noted SF6 tamponade and longer postoperative time supine were associated with 58% fewer rebubbling procedures, and an ECL not statistically different from using air. We routinely use 50% SF6 gas as some gas bubble expansion is desirable in the early postoperative period. This also aligns well with our intensive postoperative care regime of reviewing the patient one to two hours after surgery and then the day after for early release of gas/aqueous the following day and a week later. Further, we noted an overall decline in GD as we moved on from using air to SF6 in the last three years. We had one case of graft dislocation (0.9%) due to escape of SF6 gas from a leaky wound; this was included in significant GDs for analysis and is otherwise within reported dislocation rates ranging from 1.8 to 4.4% [18].

In our study, overall BCDVA at 6 months was 0.20 ± 0.28 logMAR, and endothelial cell loss was 40.46 ± 20.36%, which is comparable to previously reported figures (Table 2). Further, we noted better visual outcomes for eyes that underwent DMEK combined with cataract surgery than those already pseudophakic—this has been reported in a few studies [22]. A recent review and meta-analysis by our group also supported this [27]. Similar outcomes between the grade of the surgeon (consultant vs. fellow) and similar rates of complications (GD and rebubbling rates) emphasize the importance of following a standardized protocol [28].

To the best of our knowledge, there is only one previous study that included 31 eyes that reported outcomes of DMEK without routine PI [19]. Our case series reports clinical outcomes of PI less DMEK in 104 eyes over a 5-year period. Our study has its limitations: Firstly, it is retrospective by design. Secondly, we only included cases of Fuchs endothelial dystrophy for uniformity; hence, the results cannot be extrapolated to DMEK in other complex cases, such as previously failed grafts, complex pseudophakia, or prior glaucoma surgeries. Third, there were no eyes with phakic DMEK in our study. Lastly, the number of cases is low for a five-year period from a tertiary care eye center; this is in part due to the fact that we only included cases from a single surgeon-led team and equally in part due to the impact of the COVID-19 pandemic in 2020 and 2021. However, our study reflects real-world data of outcomes of DMEK performed using a standard protocol by surgeons of varying levels of experience at a tertiary care eye hospital. Further comparative studies or randomized trials, especially fellow eye comparison studies randomizing eyes into peripheral iridotomy versus no peripheral iridotomy may be worthwhile to compare clinical and patient-reported outcomes.

To conclude, DMEK without routine peripheral iridotomy in eyes with Fuchs endothelial dystrophy gives comparable results to the published literature for DMEK with peripheral iridotomy.

## Figures and Tables

**Table 1:** Baseline parameters of study population for PI less DMEK.

Baseline Parameters	
Uncorrected logMAR visual acuity	0.58 ± 0.36 (2 to 0.10)
Best corrected logMAR visual acuity	0.42 ± 0.35 (2 to 0.10)
Intraocular pressure (mm Hg)	15.26 ± 3.56 (8.0 to 23.6)
Donor endothelial cell density (per sq. mm)	2629 ± 159 (2300 to 3100)
Preoperative patient lens Status	Phakic = 77 (73%); Pseudophakic = 27 (27%)
Surgery performed	DMEK triple = 77 (73%); DMEK = 27 (27%)
Surgeon grade	Consultant = 64 (61%); Fellow = 40 (39%)
Tamponade agent	Air = 27 (26%); SF6 = 77 (74%)

PI—Peripheral iridotomy/iridectomy. DMEK—Descemet’s membrane endothelial keratoplasty. SF6—Sulfur hexafluoride gas.

**Table 2:** Details of all cases of pupillary block.

Case	Type of PB	Surgery and Tamponade	Description	Outcome
**1**	**Type I**	DMEK triple; air	No air release on day 0; PB on **day 1**; relieved by releasing air and aqueous from inferior paracentesis along with inferior laser PI	Mild inferior graft detachment (GD) which settled conservatively. **BCDVA 6/9** at 6 months
**2**	**Type II**	DMEK triple; SF6	SF6 released on day 0, no aqueous/SF6 released on day 1; PB noted at **day 7**, requiring laser PI and further AC reformation in theatre on day 12	Post-operative cystoid macular oedema (patient had pre-operative ERM); **BCDVA 6/12** at 6 months
**3**	**Type II**	DMEK; SF6	PB noted at **day 4**; managed by release of SF6 gas and aqueous through inferior paracentesis	Uneventful further course; **BCDVA of 6/5** at 6 months
**4**	**Type II**	DMEK triple; SF6	PB noted at **day 7**; managed by release of SF6 gas and aqueous through inferior paracentesis	Uneventful further course; **BCDVA of 6/7.5** at 6 months

PI—Peripheral iridotomy/iridectomy. DMEK—Descemet’s membrane endothelial keratoplasty. SF6—Sulfur hexafluoride gas. PB—Pupillary block. BCDVA—Best distance corrected visual acuity. Type I: bubble pushing the pupil against the lens restricting any aqueous movement through the pupil. Type II: bubble misdirection resulting in mechanical angle closure.

**Table 3:** Overview of results of DMEK in the literature.

Author, Year, Place	Protocol for Peripheral Iridotomy/Iridectomy (PI)	Sample Size and Study Design	Results				
			Pupillary Block	Graft Detachment	Rebubbling	Visual acuity (BCDVA logMAR)	Endothelial Cell Loss
Fajardo-Sanches J et al. [7] 2021 United Kingdom	Routine intra-operative inferior PI	329 eyes Retrospective comparative case series	NR	NR	17.1 to 21.1%	NR	NR
Shahnazaryan et al. [15] 2020 United Kingdom	Routine Nd:YAG laser PI before surgery.	114 eyes Retrospective comparative case series	NR	NR	2.5% (triple DMEK) 2.9% (pseudophakic DMEK)	0.00 ± 0.55 (triple DMEK) 0.04 ± 0.55 (pseudophakic DMEK)	ECL at 12 months = 41% (triple DMEK) 33% (pseudophakic DMEK)
Birbal et al. [13] 2020 Netherlands	PI at 12 o’clock using (Nd:YAG) laser two weeks before DMEK	1000 eyes Retrospective case series	NR	13%	8.2%	0.04 ± 0.10 (phakic DMEK) 0.10 ± 0.23 (pseudophakic DMEK)	ECL at 6 months = 39% (phakic DMEK) 46% (pseudophakic DMEK)
Bae et al. [12] 2020 Canada	Intra-operative PI	68 eyes Retrospective case series	NR	NR	NR	0.15 ± 0.13	NR
Schoenberg et al. [14] 2015 Indiana, USA	Intra-operative inferior PI	108 eyes Retrospective case series	NR	NR	16%	NR	29%
van Dijk et al. [16] 2016 Netherlands and USA	PI at 12 o’clock using (Nd:YAG) laser two weeks before DMEK	67 eyes Prospective case series	NR	16.4%	NR	0.07 ± 0.11	NR
Basak et al. [18] 2020 India	Intra-operative inferior PI	100 eyes Retrospective case series	2%	9%	4%	NR (average)	ECL at 3 months = 26.92 ± 13.40%
Livny et al. [19] 2018 Israel	No PI	31 eyes Retrospective case series	Zero	32%	16%	0.18±0.14	ECL up to 6 months = 49 ± 20%
Sorkin et al. [20] 2019 Canada	No PI (based on previously published technique)	45 eyes Retrospective case series	2.2%	35.6%	33.3%	0.22±0.13	ECL at 12 months = 36.5%
Parker et al. [21] 2022 Netherlands	PI using (Nd:YAG) laser 1-2 weeks before DMEK	52 eyes	11.5% (All 6 eyes had type II PB)	4%	Zero	NR	ECL at 12 months = 35.4%
von Marchtaler et al. [22] 2018 Germany	Pre-operative laser iridotomy day before DMEK	136 eyes Retrospective	2.9%	21.3%	12.5%	0.25 ± 0.15 (air) and 0.22 ± 0.16 (gas)	At 3 months: 37.3% (air) and 39.6% (SF6 gas)
Moshiri et al. [23] 2021 Germany	Preoperatively inferior YAG PI	1137 eyes Retrospective cohort study	NR	NR	52.6% (air) 13.9% (SF6 gas)	0.14 ± 0.1 (pseudophakic DMEK) 0.09 ± 0.12 (phakic DMEK) 0.12 ± 0.10 (triple DMEK)	38.54% (pseudophakic DMEK) 37.56% (phakic DMEK) 37.98% (triple DMEK)
Our results	No PI	104 eyes Retrospective	3.8%	6.6% (* Overall 43%)	3.8% (* Overall 30%)	0.20 ± 0.27 (* including all cases—graft failure and co-morbidities)	40.46 ± 20.36 at 6 months

DMEK—Descemet’s membrane endothelial keratoplasty. PI—Peripheral iridotomy/iridectomy. BCDVA—Best corrected distance visual acuity. NR—not reported. ECL—endothelial cell loss. Type I: bubble pushing the pupil against the lens restricting any aqueous movement through the pupil. Type II: bubble misdirection resulting in mechanical angle closure. (* Overall rates including mild graft detachments and air injection on slit-lamp).

## Data Availability

Data available on request from the corresponding author.
